# Decision making with visualizations: a cognitive framework across disciplines

**DOI:** 10.1186/s41235-018-0120-9

**Published:** 2018-07-11

**Authors:** Lace M. Padilla, Sarah H. Creem-Regehr, Mary Hegarty, Jeanine K. Stefanucci

**Affiliations:** 1 Northwestern University, Evanston, USA; 2 Department of Psychology, University of Utah, 380 S. 1530 E., Room 502, Salt Lake City, UT 84112 USA; 3 Department of Psychology, University of California–Santa Barbara, Santa Barbara, USA

**Keywords:** Decision making with visualizations review, Cognitive model, Visual-spatial biases, Graphs, Geospatial visualizations, Healthcare visualizations, Weather forecast visualizations, Uncertainty visualizations, Graphical decision making, Dual-process

## Abstract

Visualizations—visual representations of information, depicted in graphics—are studied by researchers in numerous ways, ranging from the study of the basic principles of creating visualizations, to the cognitive processes underlying their use, as well as how visualizations communicate complex information (such as in medical risk or spatial patterns). However, findings from different domains are rarely shared across domains though there may be domain-general principles underlying visualizations and their use. The limited cross-domain communication may be due to a lack of a unifying cognitive framework. This review aims to address this gap by proposing an integrative model that is grounded in models of visualization comprehension and a dual-process account of decision making. We review empirical studies of decision making with static two-dimensional visualizations motivated by a wide range of research goals and find significant direct and indirect support for a dual-process account of decision making with visualizations. Consistent with a dual-process model, the first type of visualization decision mechanism produces fast, easy, and computationally light decisions with visualizations. The second facilitates slower, more contemplative, and effortful decisions with visualizations. We illustrate the utility of a dual-process account of decision making with visualizations using four cross-domain findings that may constitute universal visualization principles. Further, we offer guidance for future research, including novel areas of exploration and practical recommendations for visualization designers based on cognitive theory and empirical findings.

## Significance

People use visualizations to make large-scale decisions, such as whether to evacuate a town before a hurricane strike, and more personal decisions, such as which medical treatment to undergo. Given their widespread use and social impact, researchers in many domains, including cognitive psychology, information visualization, and medical decision making, study how we make decisions with visualizations. Even though researchers continue to develop a wealth of knowledge on decision making with visualizations, there are obstacles for scientists interested in integrating findings from other domains—including the lack of a cognitive model that accurately describes decision making with visualizations. Research that does not capitalize on all relevant findings progresses slower, lacks generalizability, and may miss novel solutions and insights. Considering the importance and impact of decisions made with visualizations, it is critical that researchers have the resources to utilize cross-domain findings on this topic. This review provides a cognitive model of decision making with visualizations that can be used to synthesize multiple approaches to visualization research. Further, it offers practical recommendations for visualization designers based on the reviewed studies while deepening our understanding of the cognitive processes involved when making decisions with visualizations.

## Introduction

Every day we make numerous decisions with the aid of *visualizations*, including selecting a driving route, deciding whether to undergo a medical treatment, and comparing figures in a research paper. Visualizations are external visual representations that are systematically related to the information that they represent (Bertin, 1983; Stenning & Oberlander, 1995). The information represented might be about objects, events, or more abstract information (Hegarty, 2011). The scope of the previously mentioned examples illustrates the diversity of disciplines that have a vested interest in the influence of visualizations on decision making. While the term *decision* has a range of meanings in everyday language, here *decision making* is defined as a choice between two or more competing courses of action (Balleine, 2007).

We argue that for visualizations to be most effective, researchers need to integrate decision-making frameworks into visualization cognition research. Reviews of decision making with visual-spatial uncertainty also agree there has been a general lack of emphasis on mental processes within the visualization decision-making literature (Kinkeldey, MacEachren, Riveiro, & Schiewe, 2017; Kinkeldey, MacEachren, & Schiewe, 2014). The framework that has dominated applied decision-making research for the last 30 years is a *dual-process* account of decision making. Dual-process theories propose that we have two types of decision processes: one for automatic, easy decisions (Type 1); and another for more contemplative decisions (Type 2) (Kahneman & Frederick, 2002; Stanovich, 1999).^1^ Even though many research areas involving higher-level cognition have made significant efforts to incorporate dual-process theories (Evans, 2008), visualization research has yet to directly test the application of current decision-making frameworks or develop an effective cognitive model for decision making with visualizations. The goal of this work is to integrate a dual-process account of decision making with established cognitive frameworks of visualization comprehension.

In this paper, we present an overview of current decision-making theories and existing visualization cognition frameworks, followed by a proposal for an integrated model of decision making with visualizations, and a selective review of visualization decision-making studies to determine if there is cross-domain support for a dual-process account of decision making with visualizations. As a preview, we will illustrate Type 1 and 2 processing in decision making with visualizations using four cross-domain findings that we observed in the literature review. Our focus here is on demonstrating how dual-processing can be a useful framework for examining visualization decision-making research. We selected the cross-domain findings as relevant demonstrations of Type 1 and 2 processing that were shared across the studies reviewed, but they do not represent all possible examples of dual-processing in visualization decision-making research. The review documents each of the cross-domain findings, in turn, using examples from studies in multiple domains. These cross-domain findings differ in their reliance on Type 1 and Type 2 processing. We conclude with recommendations for future work and implications for visualization designers.

### Decision-making frameworks

Decision-making researchers have pursued two dominant research paths to study how humans make decisions under risk. The first assumes that humans make rational decisions, which are based on weighted and ordered probability functions and can be mathematically modeled (e.g. Kunz, 2004; Von Neumann, 1953). The second proposes that people often make intuitive decisions using heuristics (Gigerenzer, Todd, & ABC Research Group, 2000; Kahneman & Tversky, 1982). While there is fervent disagreement on the efficacy of heuristics and whether human behavior is rational (Vranas, 2000), there is more consensus that we can make both intuitive and strategic decisions (Epstein, Pacini, Denes-Raj, & Heier, 1996; Evans, 2008; Evans & Stanovich, 2013; cf. Keren & Schul, 2009). The capacity to make intuitive and strategic decisions is described by a *dual-process account* of decision making, which suggests that humans make fast, easy, and computationally light decisions (known as Type 1 processing) by default, but can also make slow, contemplative, and effortful decisions by employing Type 2 processing (Kahneman, 2011). Various versions of dual-processing theory exist, with the key distinctions being in the attributes associated with each type of process (for a more detailed review of dual-process theories, see Evans & Stanovich, 2013). For example, older *dual-systems* accounts of decision making suggest that each process is associated with specific cognitive or neurological systems. In contrast, dual-process (sometimes termed dual-type) theories propose that the processes are distinct but do not necessarily occur in separate cognitive or neurological systems (hence the use of process over system) (Evans & Stanovich, 2013).

**Fig. 1:**
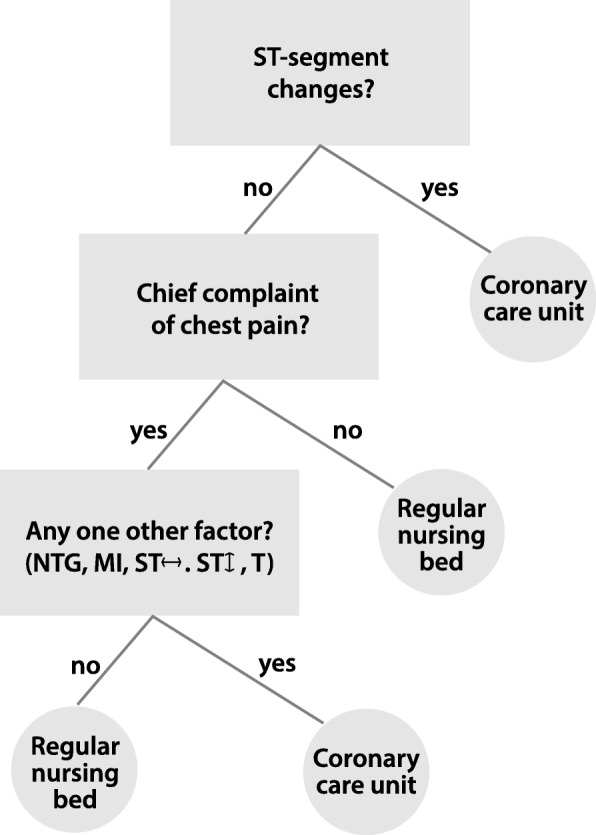
Coronary care unit decision tree, which illustrates a sequence of rules that a doctor could use to guide treatment decisions. Redrawn from “Heuristic decision making in medicine” by J. Marewski, and G. Gigerenzer 2012, *Dialogues in clinical neuroscience, 14(1)*, 77. ST-segment change refers to if certain anomaly appears in the patient’s electrocardiogram. NTG nitroglycerin, MI myocardial infarction, T T-waves with peaking or inversion

Many applied domains have adapted a dual-processing model to explain task- and domain-specific decisions, with varying degrees of success (Evans, 2008). For example, when a physician is deciding if a patient should be assigned to a coronary care unit or a regular nursing bed, the doctor can use a heuristic or utilize heart disease predictive instruments to make the decision (Marewski & Gigerenzer, 2012). In the case of the heuristic, the doctor would employ a few simple rules (diagrammed in Fig. 1) that would guide her decision, such as considering the patient’s chief complaint being chest pain. Another approach is to apply deliberate mental effort to make a more time-consuming and effortful decision, which could include using heart disease predictive instruments (Marewski & Gigerenzer, 2012). In a review of how applied domains in higher-level cognition have implemented a dual-processing model for domain-specific decisions, Evans (2008) argues that prior work has conflicting accounts of Type 1 and 2 processing. Some studies suggest that the two types work in parallel while others reveal conflicts between the Types (Sloman, 2002). In the physician example proposed by Marewski and Gigerenzer (2012), the two types are not mutually exclusive, as doctors can utilize Type 2 to make a more thoughtful decision that is also influenced by some rules of thumb or Type 1. In sum, Evans (2008) argues that due to the inconsistency of classifying Type 1 and 2, the distinction between only two types is likely an oversimplification. Evans (2008) suggests that the literature only consistently supports the identification of processes that require a capacity-limited, working memory resource versus those that do not. Evans and Stanovich (2013) updated their definition based on new behavioral and neuroscience evidence stating, “the defining characteristic of Type 1 processes is their autonomy. They do not require ‘controlled attention,’ which is another way of saying that they make minimal demands on working memory resources” (p. 236). There is also debate on how to define the term *working memory* (Cowan, 2017). In line with prior work on decision making with visualizations (Patterson et al., 2014), we adopt the definition that working memory consists of multiple components that maintain a limited amount of information (their capacity) for a finite period (Cowan, 2017). Contemporary theories of *working memory* also stress the ability to engage attention in a controlled manner to suppress automatic responses and maintain the most task-relevant information with limited capacity (Engle, Kane, & Tuholski, 1999; Kane, Bleckley, Conway, & Engle, 2001; Shipstead, Harrison, & Engle, 2015).

Identifying processes that require significant working memory provides a definition of Type 2 processing with observable neural correlates. Therefore, in line with Evans and Stanovich (2013), in the remainder of this manuscript, we will use significant working memory capacity demands and significant need for cognitive control, as defined above, as the criterion for Type 2 processing. In the context of visualization decision making, processes that require significant working memory are those that depend on the deliberate application of working memory to function. Type 1 processing occurs outside of users’ conscious awareness and may utilize small amounts of working memory but does not rely on conscious processing in working memory to drive the process. It should be noted that Type 1 and 2 processing are not mutually exclusive and many real-world decisions likely incorporate all processes. This review will attempt to identify tasks in visualization decision making that require significant working memory and capacity (Type 2 processing) and those that rely more heavily on Type 1 processing, as a first step to combining decision theory with visualization cognition.

### Visualization cognition

**Fig. 2:**
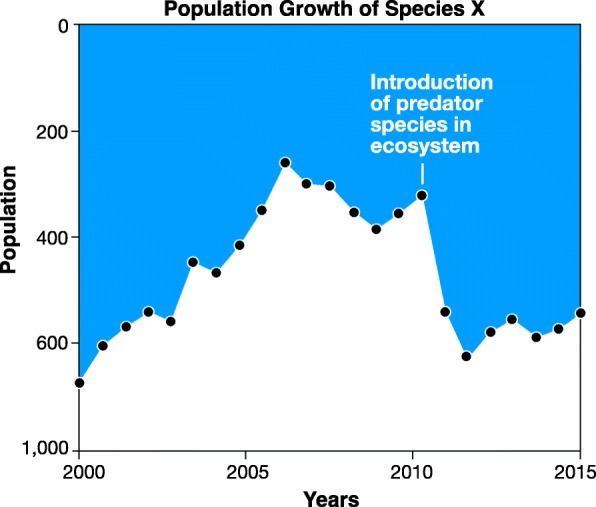
Fictional relationship between the population growth of Species X and a predator species, where the *Y-axis* ordering does not match standard graphic conventions. Notice that the *y-axis* is reverse ordered. This figure was inspired by a controversial graphic produced by Christine Chan of Reuters, which showed the relationship between Florida’s “Stand Your Ground” law and firearm murders with the Y-axis reversed ordered (Lallanilla, 2014)

Visualization cognition is a subset of *visuospatial* reasoning, which involves deriving meaning from external representations of visual information that maintain consistent spatial relations (Tversky, 2005). Broadly, two distinct approaches delineate visualization cognition models (Shah, Freedman, & Vekiri, 2005). The first approach refers to perceptually focused frameworks which attempt to specify the processes involved in perceiving visual information in displays and make predictions about the speed and efficiency of acquiring information from a visualization (e.g. Hollands & Spence, 1992; Lohse, 1993; Meyer, 2000; Simkin & Hastie, 1987). The second approach considers the influence of prior knowledge as well as perception. For example, *Cognitive Fit Theory* (Vessey, 1991), suggests that the user compares a learned graphic convention (mental schema) to the visual depiction. Visualizations that do not match the mental schema require cognitive transformations to make the visualization and mental representation align. For example, Fig. 2 illustrates a fictional relationship between the population growth of Species X and a predator species. At first glance, it may appear that when the predator species was introduced that the population of Species X dropped. However, after careful observation, you may notice that the *higher* population values are located *lower* on the Y-axis, which does not match our mental schema for graphs. With some effort, you can mentally reorder the values on the Y-axis to match your mental schema and then you may notice that the introduction of the predator species actually correlates with *growth* in the population of Species X. When the viewer is forced to mentally transform the visualization to match their mental schema, processing steps are increased, which may increase errors, time to complete a task, and demand on working memory (Vessey, 1991).

**Fig. 3:**
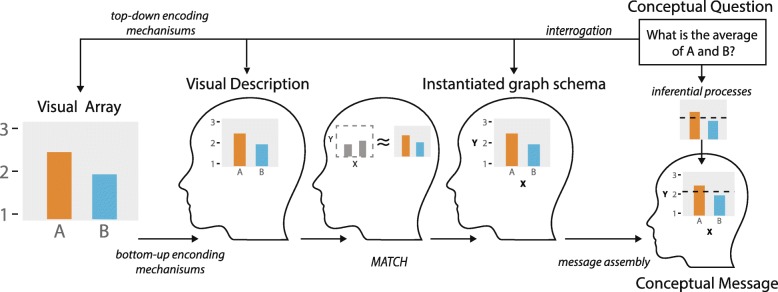
Adapted figure from the Pinker (1990) model of visualization comprehension, which illustrates each process

Pinker (1990) proposed a cognitive model (see Fig. 3), which provides an integrative structure that denotes the distinction between top-down and bottom-up encoding mechanisms in understanding data graphs. Researchers have generalized this model to propose theories of comprehension, learning, and memory with visual information (Hegarty, 2011; Kriz & Hegarty, 2007; Shah & Freedman, 2011). The Pinker (1990) model suggests that from the *visual array*, defined as the unprocessed neuronal firing in response to visualizations, bottom-up encoding mechanisms are utilized to construct a *visual description*, which is the mental encoding of the visual stimulus. Following encoding, viewers mentally search long-term memory for knowledge relevant for interpreting the visualization. This knowledge is proposed to be in the form of a graph schema.

Then viewers use a *match* process, where the graph schema that is the most similar to the visual array is retrieved. When a matching graph schema is found, the schema becomes *instantiated*. The visualization conventions associated with the graph schema can then help the viewer interpret the visualization (*message assembly* process). For example, Fig. 3 illustrates comprehension of a bar chart using the Pinker (1990) model. In this example, the matched graph schema for a bar graph specifies that the dependent variable is on the Y-axis and the independent variable is on the X-axis; the instantiated graph schema incorporates the visual description and this additional information. The *conceptual message* is the resulting mental representation of the visualization that includes all supplemental information from long-term memory and any mental transformations the viewer may perform on the visualization. Viewers may need to transform their mental representation of the visualization based on their task or *conceptual question*. In this example, the viewer’s task is to find the average of A and B. To do this, the viewer must interpolate information in the bar chart and update the conceptual message with this additional information. The conceptual question can guide the construction of the mental representation through *interrogation*, which is the process of seeking out information that is necessary to answer the conceptual question. Top-down encoding mechanisms can influence each of the processes.

The influences of top-down processes are also emphasized in a previous attempt by Patterson et al. (2014) to extend visualization cognition theories to decision making. The Patterson et al. (2014) model illustrates how top-down cognitive processing influences encoding, pattern recognition, and working memory, but not decision making or the response. Patterson et al. (2014) use the multicomponent definition of working memory, proposed by Baddeley and Hitch (1974) and summarized by Cowan (2017) as a “multicomponent system that holds information temporarily and mediates its use in ongoing mental activities” (p. 1160). In this conception of working memory, a *central executive* controls the functions of working memory. The central executive can, among other functions, control attention and hold information in a *visuo-spatial temporary store*, which is where information can be maintained temporally for decision making without being stored in long-term memory (Baddeley & Hitch, 1974).

While incorporating working memory into a visualization decision-making model is valuable, the Patterson et al. (2014) model leaves some open questions about relationships between components and processes. For example, their model lacks a pathway for working memory to influence decisions based on top-down processing, which is inconsistent with well-established research in decision science (e.g. Gigerenzer & Todd, 1999; Kahneman & Tversky, 1982). Additionally, the *normal* processing pathway, depicted in the Patterson model, is an oversimplification of the interaction between top-down and bottom-up processing that is documented in a large body of literature (e.g. Engel, Fries, & Singer, 2001; Mechelli, Price, Friston, & Ishai, 2004).

### A proposed integrated model of decision making with visualizations

**Fig. 4:**
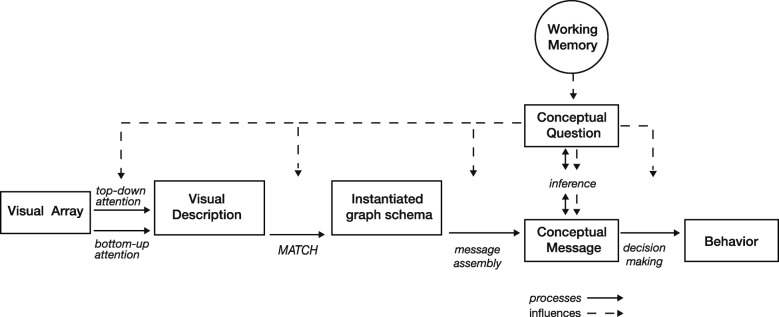
Model of visualization decision making, which emphasizes the influence of working memory. Long-term memory can influence all components and processes in the model either via pre-attentive processes or by conscious application of knowledge

**Fig. 5:**
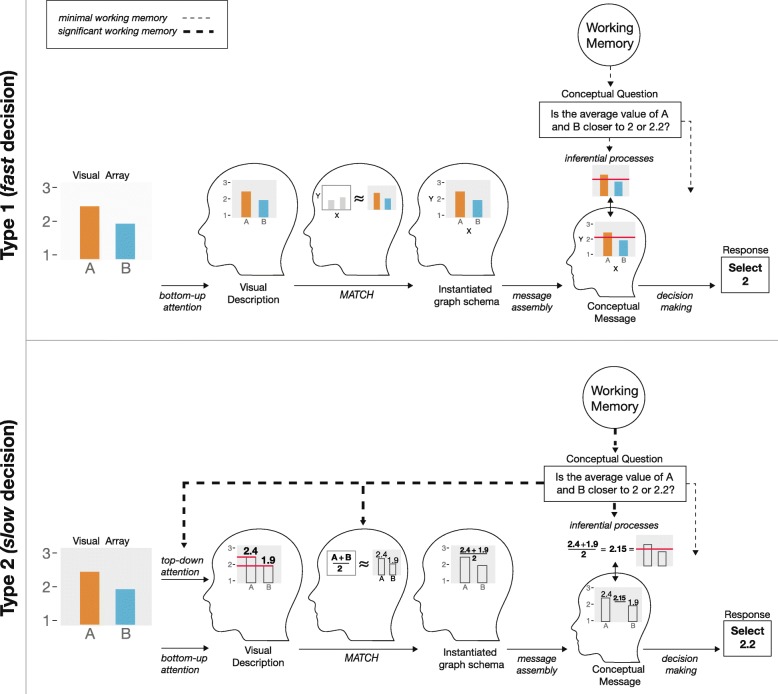
Examples of a fast Type 1 (top) and slow Type 2 (bottom) decision outlined in our proposed model of decision making with visualizations. In these examples, the viewer’s task is to decide if the average value of bars A and B are closer to 2 or 2.2. The thick dotted line denotes significant working memory and the thin dotted line negligible working memory

Our proposed model (Fig. 4) introduces a dual-process account of decision making (Evans & Stanovich, 2013; Gigerenzer & Gaissmaier, 2011; Kahneman, 2011) into the Pinker (1990) model of visualization comprehension. A primary addition of our model is the inclusion of working memory, which is utilized to answer the conceptual question and could have a subsequent impact on each stage of the decision-making process, except bottom-up attention. The final stage of our model includes a decision-making process that derives from the conceptual message and informs behavior. In line with a dual-process account (Evans & Stanovich, 2013; Gigerenzer & Gaissmaier, 2011; Kahneman, 2011), the decision step can either be completed with Type 1 processing, which only uses minimal working memory (Evans & Stanovich, 2013) or recruit significant working memory, constituting Type 2 processing. Also following Evans and Stanovich (2013), we argue that people can make a decision with a visualization while using minimal amounts of working memory. We classify this as Type 1 thinking. Lohse (1997) found that when participants made judgments about budget allocation using profit charts, individuals with less working memory capacity performed equally well compared to those with more working memory capacity, when they only made decisions about three regions (easier task). However, when participants made judgments about nine regions (harder task), individuals with more working memory capacity outperformed those with less working memory capacity. The results of the study reveal that individual differences in working memory capacity only influence performance on complex decision-making tasks (Lohse, 1997). Figure 5 (top) illustrates one way that a viewer could make a Type 1 decision about whether the average value of bars A and B is closer to 2 or 2.2. Figure 5 (top) illustrates how a viewer might make a fast and computationally light decision in which she decides that the middle point between the two bars is closer to the salient tick mark of 2 on the Y-axis and answers 2 (which is incorrect). In contrast, Fig. 5 (bottom) shows a second possible method of solving the same problem by utilizing significant working memory (Type 2 processing). In this example, the viewer has recently learned a strategy to address similar problems, uses working memory to guide a top-down attentional search of the visual array, and identifies the values of A and B. Next, she instantiates a different graph schema than in the prior example by utilizing working memory and completes an effortful mental computation of 2.4 + 1.9/2. Ultimately, the application of working memory leads to a different and more effortful decision than in Fig. 5 (top). This example illustrates how significant amounts of working memory can be used at early stages of the decision-making process and produce downstream effects and more considered responses. In the following sections, we provide a selective review of work on decision making with visualizations that demonstrates direct and indirect evidence for our proposed model.

## Empirical studies of visualization decision making

### Review method

To determine if there is cross-domain empirical support for a dual-process account of decision making with visualizations, we selectively reviewed studies of complex decision making with computer-generated two-dimensional (2D) static visualizations. To illustrate the application of a dual-process account of decision making to visualization research, this review highlights representative studies from diverse application areas. Interdisciplinary groups conducted many of these studies and, as such, it is not accurate to classify the studies in a single discipline. However, to help the reader evaluate the cross-domain nature of these findings, Table 1 includes the application area for the specific tasks used in each study.

**Table 1:** Application area for the tasks used in the reviewed studies

Task application area	Studies
Meteorology, weather, and natural disaster forecasting, weather communication	Cheong et al. (2016); Fabrikant et al. (2010); Gattis and Holyoak (1996); Hegarty et al. (2010); Joslyn and LeClerc (2013); Padilla et al. (2015); Ruginski et al. (2016)
Health research, medical images, health risk communication	Ancker et al. (2006); Fagerlin et al. (2005); Garcia-Retamero and Galesic (2009); Keehner et al. (2011); Keller et al. (2009); McCabe and Castel (2008); Okan et al. (2015); Okan, Garcia-Retamero, Cokely, and Maldonado (2012); Schirillo and Stone (2005); Stone et al. (2003); Stone et al. (1997); Waters et al. (2006); Waters et al. (2007)
Land-use planning, spatial planning, urban planning	Dennis and Carte (1998); Lee and Bednarz (2009); Smelcer and Carmel (1997); Wilkening and Fabrikant (2011)
Cost comparison, finance	Lohse (1993); Vessey and Galletta (1991)
Geospatial location	Hegarty et al. (2016); McKenzie et al. (2016)
Error-bar interpretation, graph comparison, statistics communication, science reasoning	Belia et al. (2005); Feeney et al. (2000); Newman and Scholl (2012); Sanchez and Wiley (2006); Wainer et al. (1999)
Map reading, map perception	Brügger et al. (2017); St. John et al. (2001)
Social network, computer connections	Tversky et al. (2012); Zhu and Watts (2010)
Map-based threat identification, emergency management	Bailey et al. (2007); Shen et al. (2012)

In reviewing this work, we observed four key cross-domain findings that support a dual-process account of decision making (see Table 2). The first two support the inclusion of Type 1 processing, which is illustrated by the direct path for bottom-up attention to guide decision making with the minimal application of working memory (see Fig. 5 top). The first finding is that visualizations direct viewers’ *bottom-up attention*, which can both help and hinder decision making (see “Bottom-up attention”). The second finding is that *visual-spatial biases* comprise a unique category of bias that is a direct result of the visual encoding technique (see “Visual-Spatial Biases”). The third finding supports the inclusion of Type 2 processing in our proposed model and suggests that visualizations vary in *cognitive fit* between the visual description, graph schema, and conceptual question. If the fit is poor (i.e. there is a mismatch between the visualization and a decision-making component), working memory is used to perform corrective mental transformations (see “Cognitive fit”). The final cross-domain finding proposes that *knowledge-driven processes* may interact with the effects of the visual encoding technique (see “Knowledge-driven processing”) and could be a function of either Type 1 or 2 processes. Each of these findings will be detailed at length in the relevant sections. The four cross-domain findings do not represent an exhaustive list of all cross-domain findings that pertain to visualization cognition. However, these were selected as illustrative examples of Type 1 and 2 processing that include significant contributions from multiple domains. Further, some of the studies could fit into multiple sections and were included in a particular section as illustrative examples.

**Table 2:** Overview of the four cross-domain findings along with the type of processing that they reflect

		Evidence for Type		
	Cross-domain finding	1	2	Either
1	Visualizations direct viewers’ *bottom-up attention*, which can both help and hinder decision making.	×		
2	The visual encoding technique gives rise to *visual-spatial biases*.	×		
3	Visualizations that have greater *cognitive fit* produce faster and more effective decisions.		×	
4	*Knowledge-driven processes* can interact with the effects of the encoding technique.			×

The italicised words correspond to section titles

### Type 1

#### Bottom-up attention

The first cross-domain finding that characterizes Type 1 processing in visualization decision making is that visualizations direct participants’ *bottom-up attention* to specific visual features, which can be either beneficial or detrimental to decision making. Bottom-up attention consists of involuntary shifts in focus to salient features of a visualization and does not utilize working memory (Connor, Egeth, & Yantis, 2004), therefore it is a Type 1 process. The research reviewed in this section illustrates that bottom-up attention has a profound influence on decision making with visualizations. A summary of visual features that studies have used to attract bottom-up attention can be found in Table 3.

**Table 3:** Visual features used in the reviewed studies to attract bottom-up attention

Features	Studies
Color	Fabrikant et al. (2010); Hegarty et al. (2010)
Edges and lines	Fabrikant et al. (2010); Hegarty et al. (2010); Padilla, Ruginski, and Creem-Regehr (2017)
Foreground information	Schirillo and Stone (2005); Stone et al. (2003); Stone et al. (1997)

**Fig. 6:**
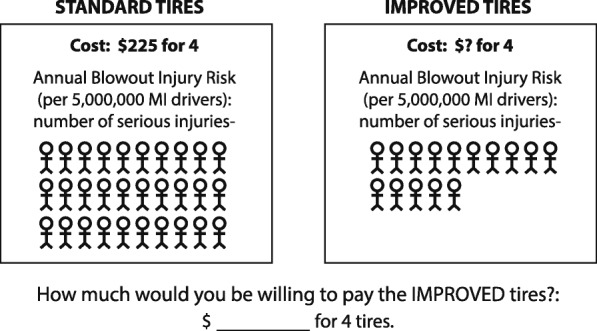
Icon arrays used to illustrate the risk of standard or improved tires. Participants were tasked with deciding how much they would pay for the improved tires. Note the base rate of 5 M drivers was represented in text. Redrawn from “Effects of numerical and graphical displays on professed risk-taking behavior” by E. R. Stone, J. F. Yates, & A. M. Parker. 1997, *Journal of Experimental Psychology: Applied*, *3*(4), 243

Numerous studies show that salient information in a visualization draws viewers’ attention (Fabrikant, Hespanha, & Hegarty, 2010; Hegarty, Canham, & Fabrikant, 2010; Hegarty, Friedman, Boone, & Barrett, 2016; Padilla, Ruginski, & Creem-Regehr, 2017; Schirillo & Stone, 2005; Stone et al., 2003; Stone, Yates, & Parker, 1997). The most common methods for demonstrating that visualizations focus viewers’ attention is by showing that viewers miss non-salient but task-relevant information (Schirillo & Stone, 2005; Stone et al., 1997; Stone et al., 2003), viewers are biased by salient information (Hegarty et al., 2016; Padilla, Ruginski et al., 2017) or viewers spend more time looking at salient information in a visualization (Fabrikant et al., 2010; Hegarty et al., 2010). For example, Stone et al. (1997) demonstrated that when viewers are asked how much they would pay for an improved product using the visualizations in Fig. 6, they focus on the number of icons while missing the base rate of 5,000,000. If a viewer simply totals the icons, the standard product appears to be twice as dangerous as the improved product, but because the base rate is large, the actual difference between the two products is insignificantly small (0.0000003; Stone et al., 1997). In one experiment, participants were willing to pay $125 more for improved tires when viewing the visualizations in Fig. 6 compared to a purely textual representation of the information. The authors also demonstrated the same effect for improved toothpaste, with participants paying $0.95 more when viewing a visual depiction compared to text. The authors’ term this heuristic of focusing on salient information and ignoring other data the *foreground effect* (Stone et al., 1997) (see also Schirillo & Stone, 2005; Stone et al., 2003).

**Fig. 7:**
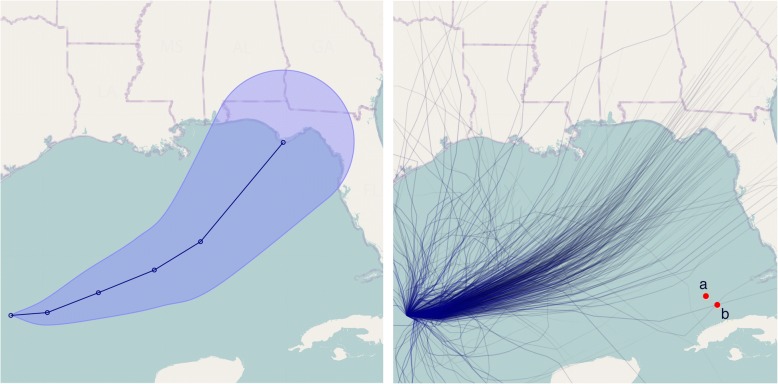
An example of the Cone of Uncertainty (*left*) and the same data represented as hurricane paths (*right*). Participants were tasked with evaluating the level of damage that would incur to offshore oil rigs at specific locations, based on the hurricane forecast visualization. Redrawn from “Effects of ensemble and summary displays on interpretations of geospatial uncertainty data” by L. M. Padilla, I. Ruginski, and S. H. Creem-Regehr. 2017, *Cognitive Research: Principles and Implications*, *2*(1), 40

A more direct test of visualizations guiding bottom-up attention is to examine if salient information biases viewers’ judgments. One method involves identifying salient features using a behaviorally validated saliency model, which predicts the locations that will attract viewers’ bottom-up attention (Harel, 2015; Itti, Koch, & Niebur, 1998; Rosenholtz & Jin, 2005). In one study, researchers compared participants’ judgments with different hurricane forecast visualizations and then, using the Itti et al. (1998) saliency algorithm, found that the differences in what was salient in the two visualizations correlated with participants’ performance (Padilla, Ruginski et al., 2017). Specifically, they suggested that the salient borders of the *Cone of Uncertainty* (see Fig. 7, left), which is used by the National Hurricane Center to display hurricane track forecasts, leads some people to incorrectly believe that the hurricane is growing in physical size, which is a misunderstanding of the probability distribution of hurricane paths that the cone is intended to represent (Padilla, Ruginski et al., 2017; see also Ruginski et al., 2016). Further, they found that when the same data were represented as individual hurricane paths, such that there was no salient boundary (see Fig. 7, right), viewers intuited the probability of hurricane paths more effectively than the Cone of Uncertainty. However, an individual hurricane path biased viewers’ judgments if it intersected a point of interest. For example, in Fig. 7 (right), participants accurately judged that locations closer to the densely populated lines (highest likelihood of storm path) would receive more damage. This correct judgment changed when a location farther from the center of the storm was intersected by a path, but the closer location was not (see locations a and b in Fig. 7 right). With both visualizations, the researchers found that viewers were negatively biased by the salient features for some tasks (Padilla, Ruginski et al., 2017; Ruginski et al., 2016).

**Fig. 8:**
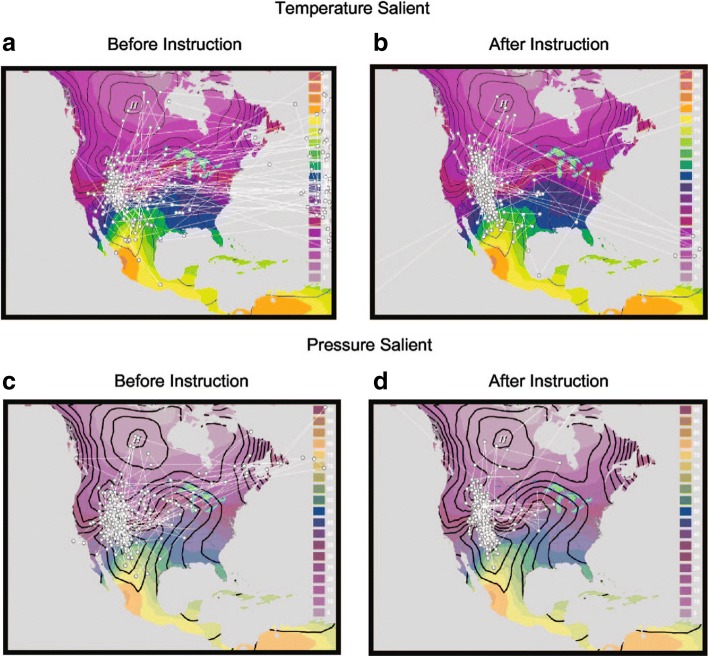
Eye-tracking data from Hegarty et al. (2010). Participants viewed an arrow located in Utah (obscured by eye-tracking data in the figure) and made judgments about whether the arrow correctly identified the wind direction. The *black isobars* were the task-relevant information. Notice that after instructions, viewers with the pressure-salient visualizations focused on the isobars surrounding Utah, rather than on the legend or in other regions. The panels correspond to the conditions in the original study

That is not to say that saliency only negatively impacts decisions. When incorporated into visualization design, saliency can guide bottom-up attention to task-relevant information, thereby improving performance (e.g. Fabrikant et al., 2010; Fagerlin, Wang, & Ubel, 2005; Hegarty et al., 2010; Schirillo & Stone, 2005; Stone et al., 2003; Waters, Weinstein, Colditz, & Emmons, 2007). One compelling example using both eye-tracking measures and a saliency algorithm demonstrated that salient features of weather maps directed viewers’ attention to different variables that were visualized on the maps (Hegarty et al., 2010) (see also Fabrikant et al., 2010). Interestingly, when the researchers manipulated the relative salience of temperature versus pressure (see Fig. 8), the salient features captured viewers’ overt attention (as measured by eye fixations) but did *not* influence performance, until participants were trained on how to effectively interpret the features. Once viewers were trained, their judgments were facilitated when the relevant features were more salient (Hegarty et al., 2010). This is an instructive example of how saliency may direct viewers’ bottom-up attention but may not influence their performance until viewers have the relevant top-down knowledge to capitalize on the affordances of the visualization.

In sum, the reviewed studies suggest that bottom-up attention has a profound influence on decision making with visualizations. This is noteworthy because bottom-up attention is a Type 1 process. At a minimum, the work suggests that Type 1 processing influences the first stages of decision making with visualizations. Further, the studies cited in this section provide support for the inclusion of bottom-up attention in our proposed model.

#### Visual-spatial biases

A second cross-domain finding that relates to Type 1 processing is that visualizations can give rise to *visual-spatial biases* that can be either beneficial or detrimental to decision making. We are proposing the new concept of visual-spatial biases and defining this term as a bias that elicits heuristics, which are a direct result of the visual encoding technique. Visual-spatial biases likely originate as a Type 1 process as we suspect they are connected to bottom-up attention, and if detrimental to decision making, have to be actively suppressed by top-down knowledge and cognitive control mechanisms (see Table 4 for summary of biases documented in this section). Visual-spatial biases can also improve decision-making performance. As Card, Mackinlay, and Shneiderman (1999) point out, we can use *vision to think*, meaning that visualizations can capitalize on visual perception to interpret a visualization without effort when the visual biases elucidated by the visualization are consistent with the correct interpretation.

**Table 4:** Biases documented in the reviewed studies

Bias	Studies
Anchoring	Belia et al. (2005)
Anecdotal evidence	Fagerlin et al. (2005)
Containment	McKenzie et al. (2016); Joslyn and LeClerc (2013); Grounds et al. (2017); Newman and Scholl (2012); Ruginski et al. (2016)
Deterministic construal	Grounds et al. (2017); Joslyn and LeClerc (2013)
High-quality image	Keehner et al. (2011); McCabe and Castel (2008); St. John et al. (2001); Ancker et al. (2006); Brügger et al. (2017); Hegarty et al. (2012); Wainer et al. (1999); Wilkening and Fabrikant (2011)
Risk aversion	Schirillo and Stone (2005)
Side effect aversion	Waters et al. (2006); Waters et al. (2007)

**Fig. 9:**
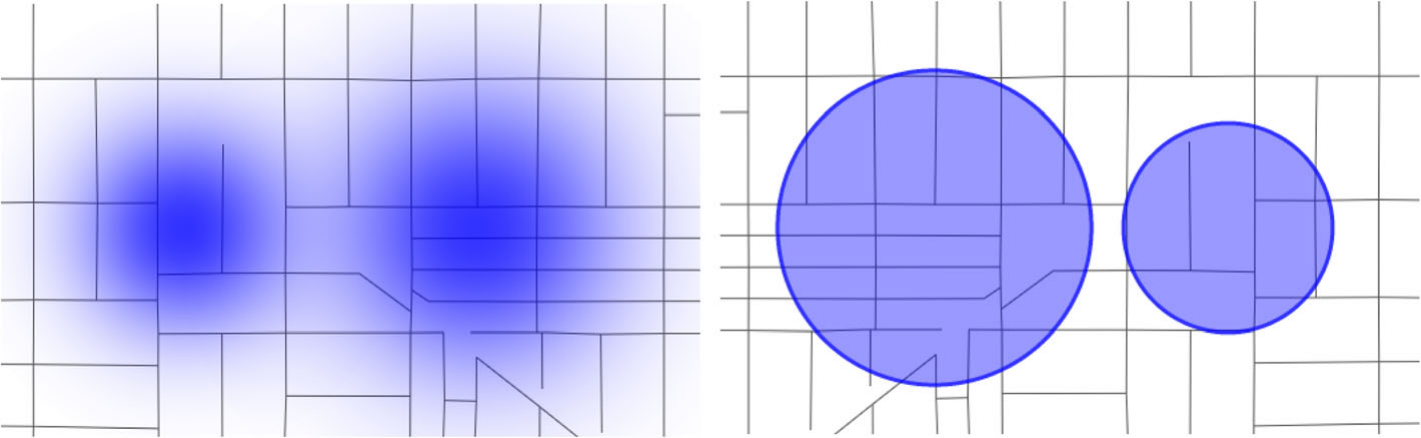
Example stimuli from McKenzie et al. (2016) showing circular semi-transparent overlays used by Google Maps to indicate the uncertainty of the users’ location. Participants compared two versions of these visualizations and determined which represented the most accurate positional location. Redrawn from “Assessing the effectiveness of different visualizations for judgments of positional uncertainty” by G. McKenzie, M. Hegarty, T. Barrett, and M. Goodchild. 2016, *International Journal of Geographical Information Science*, *30*(2), 221–239

Tversky (2011) presents a taxonomy of visual-spatial communications that are intrinsically related to thought, which are likely the bases for visual-spatial biases (see also Fabrikant & Skupin, 2005). One of the most commonly documented visual-spatial biases that we observed across domains is a containment conceptualization of boundary representations in visualizations. Tversky (2011) makes the analogy, “Framing a picture is a way of saying that what is inside the picture has a different status from what is outside the picture” (p. 522). Similarly, Fabrikant and Skupin (2005) describe how, “They [boundaries] help partition an information space into zones of relative semantic homogeneity” (p. 673). However, in visualization design, it is common to take *continuous* data and visually represent them with boundaries (i.e. summary statistics, error bars, isocontours, or regions of interest; Padilla et al., 2015; Padilla, Quinan, Meyer, & Creem-Regehr, 2017). Binning continuous data is a reasonable approach, particularly when intended to make the data simpler for viewers to understand (Padilla, Quinan, et al., 2017). However, it may have the unintended consequence of creating artificial boundaries that can bias users—leading them to respond as if data within a containment is more similar than data across boundaries. For example, McKenzie, Hegarty, Barrett, and Goodchild (2016) showed that participants were more likely to use a containment heuristic to make decisions about Google Map’s blue dot visualization when the positional uncertainty data were visualized as a bounded circle (Fig. 9 right) compared to a Gaussian fade (Fig. 9 left) (see also Newman & Scholl, 2012; Ruginski et al., 2016). Recent work by Grounds, Joslyn, and Otsuka (2017) found that viewers demonstrate a “deterministic construal error” or the belief that visualizations of temperature uncertainty represent a deterministic forecast. However, the deterministic construal error was not observed with textual representations of the same data (see also Joslyn & LeClerc, 2013).

**Fig. 10:**
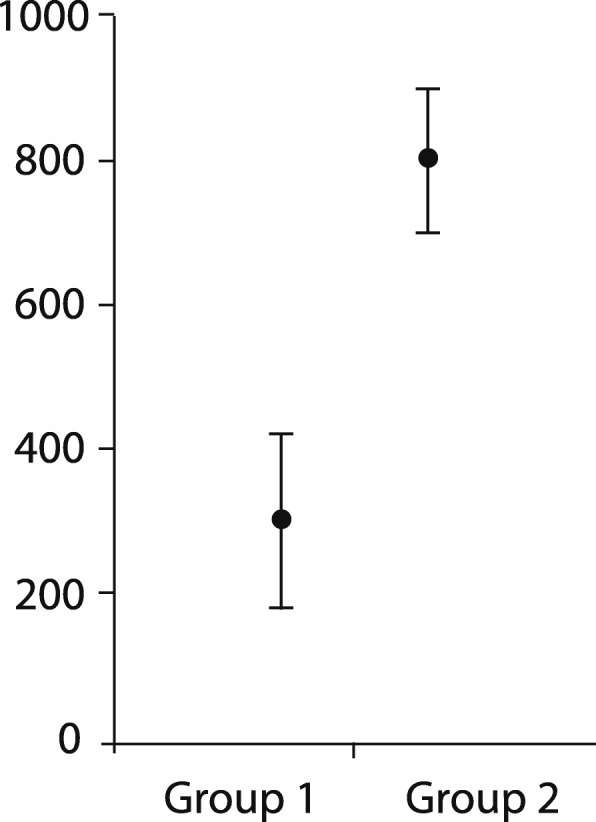
Example display and instructions from Belia et al. (2005). Redrawn from “Researchers misunderstand confidence intervals and standard error bars” by S. Belia, F. Fidler, J. Williams, and G. Cumming. 2005, *Psychological Methods, 10*(4), 390. Copyright 2005 by “American Psychological Association”

Additionally, some visual-spatial biases follow the same principles as more well-known decision-making biases revealed by researchers in behavioral economics and decision science. In fact, some decision-making biases, such as *anchoring*, the tendency to use the first data point to make relative judgments, seem to have visual correlates (Belia, Fidler, Williams, & Cumming, 2005). For example, Belia et al. (2005) asked experts with experience in statistics to align two means (representing “Group 1” and “Group 2”) with error bars so that they represented data ranges that were just significantly different (see Fig. 10 for example of stimuli). They found that when the starting position of Group 2 was around 800 ms, participants placed Group 2 higher than when the starting position for Group 2 was at around 300 ms. This work demonstrates that participants used the starting mean of Group 2 as an anchor or starting point of reference, even though the starting position was arbitrary. Other work finds that visualizations can be used to reduce some decision-making biases including anecdotal evidence bias (Fagerlin et al., 2005), side effect aversion (Waters et al., 2007; Waters, Weinstein, Colditz, & Emmons, 2006), and risk aversion (Schirillo & Stone, 2005).

**Fig. 11:**
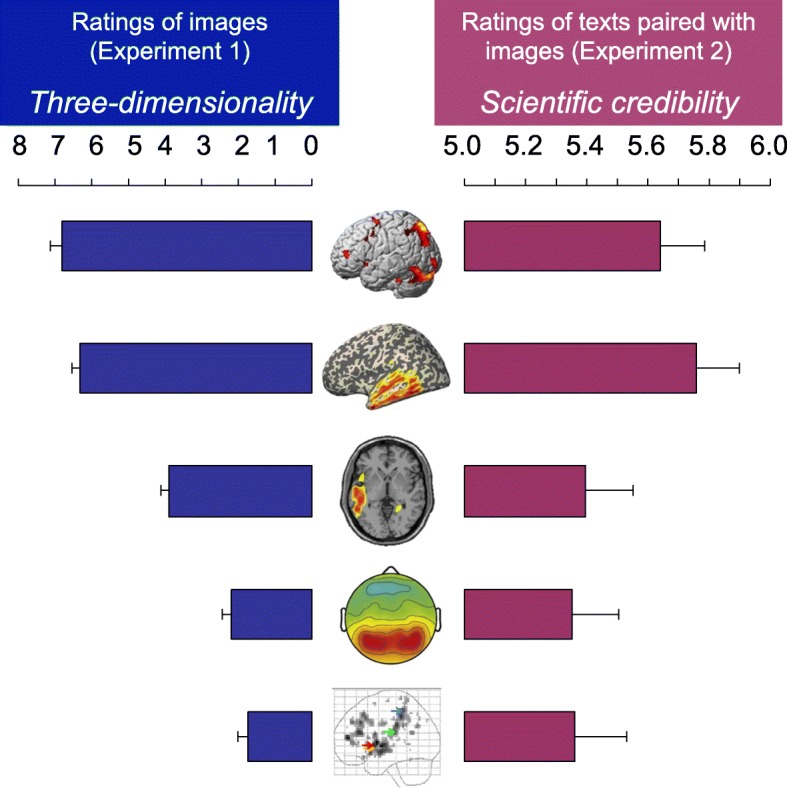
Image showing participants’ ratings of three-dimensionality and scientific credibility for a given neuroimaging visualization, originally published in grayscale (Keehner et al., 2011)

**Fig. 12:**
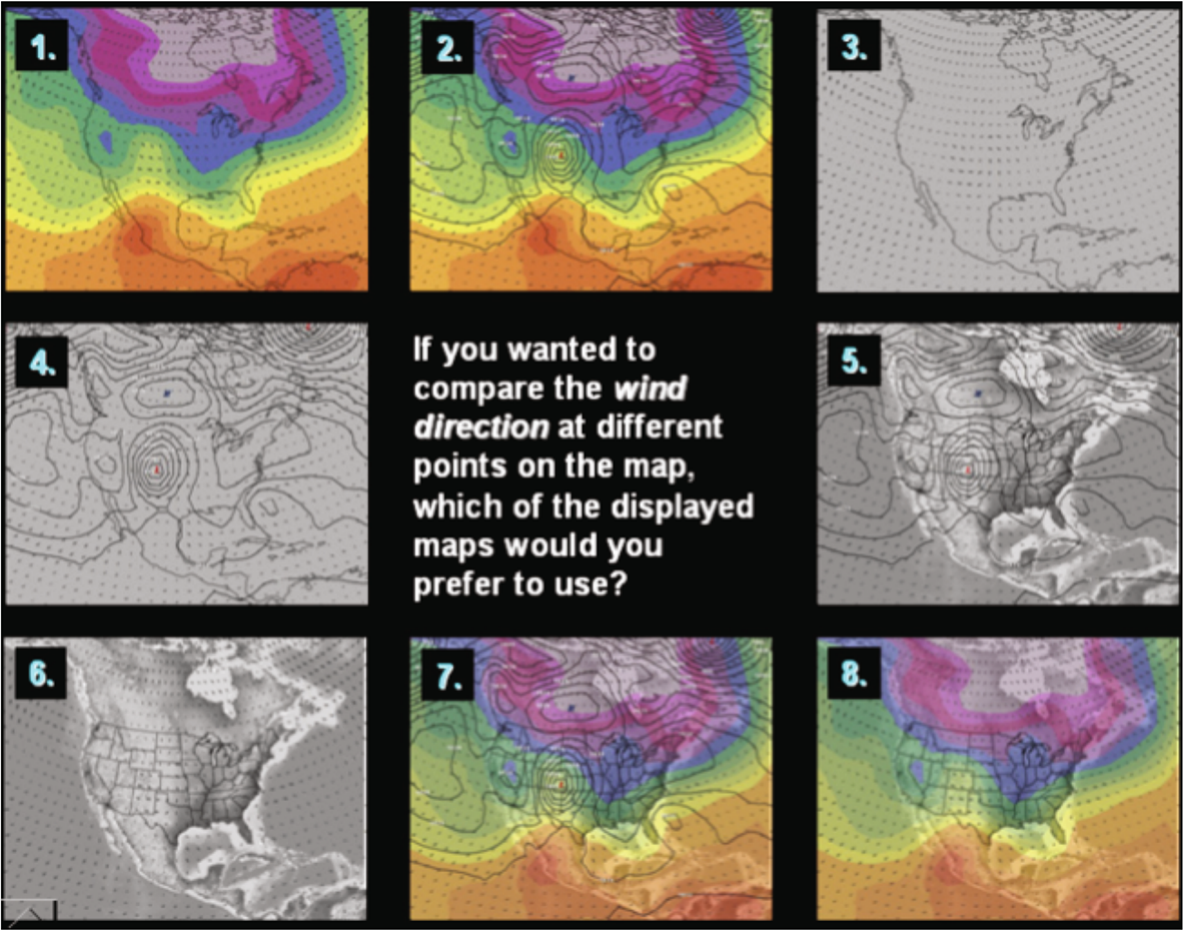
Example stimuli from Hegarty et al. (2012) showing maps with varying levels of realism. Both novice viewers and meteorologists were tasked with selecting a visualization to use and performing a geospatial task. The panels correspond to the conditions in the original study

Additionally, the mere presence of a visualization may inherently bias viewers. For example, viewers find scientific articles with high-quality neuroimaging figures to have greater scientific reasoning than the same article with a bar chart or without a figure (McCabe & Castel, 2008). People tend to unconsciously believe that high-quality scientific images reflect high-quality science—as illustrated by work from Keehner, Mayberry, and Fischer (2011) showing that viewers rate articles with three-dimensional brain images as more scientific than those with 2D images, schematic drawings, or diagrams (See Fig. 11). Unintuitively, however, high-quality complex images can be detrimental to performance compared to simpler visualizations (Hegarty, Smallman, & Stull, 2012; St. John, Cowen, Smallman, & Oonk, 2001; Wilkening & Fabrikant, 2011). Hegarty et al. (2012) demonstrated that novice users prefer realistically depicted maps (see Fig. 12), even though these maps increased the time taken to complete the task and focused participants’ attention on irrelevant information (Ancker, Senathirajah, Kukafka, & Starren, 2006; Brügger, Fabrikant, & Çöltekin, 2017; St. John et al., 2001; Wainer, Hambleton, & Meara, 1999; Wilkening & Fabrikant, 2011). Interestingly, professional meteorologists also demonstrated the same biases as novice viewers (Hegarty et al., 2012) (see also Nadav-Greenberg, Joslyn, & Taing, 2008).

We argue that visual-spatial biases reflect a Type 1 process, occurring automatically with minimal working memory. Work by Sanchez and Wiley (2006) provides direct evidence for this assertion using eye-tracking data to demonstrate that individuals with less working memory capacity attend to irrelevant images in a scientific article more than those with greater working memory capacity. The authors argue that we are naturally drawn to images (particularly high-quality depictions) and that significant working memory capacity is required to shift focus away from images that are task-irrelevant. The ease by which visualizations captivate our focus and direct our bottom-up attention to specific features likely increases the impact of these biases, which may be why some visual-spatial biases are notoriously difficult to override using working memory capacity (see Belia et al., 2005; Boone, Gunalp, & Hegarty, in press; Joslyn & LeClerc, 2013; Newman & Scholl, 2012). We speculate that some visual-spatial biases are intertwined with bottom-up attention—occurring early in the decision-making process and influencing the down-stream processes (see our model in Fig. 4 for reference), making them particularly unremitting.

### Type 2

#### Cognitive fit

We also observe a cross-domain finding involving Type 2 processing, which suggests that if there is a mismatch between the visualization and a decision-making component, working memory is used to perform corrective mental transformations. *Cognitive fit* is a term used to describe the correspondence between the visualization and conceptual question or task (see our model for reference; for an overview of cognitive fit, see Vessey, Zhang, & Galletta, 2006). Those interested in examining cognitive fit generally attempt to identify and reduce mismatches between the visualization and one of the decision-making components (see Table 5 for a breakdown of the decision-making components that the reviewed studies evaluated). When there is a mismatch produced by the default Type 1 processing, it is argued that significant working memory (Type 2 processing) is required to resolve the discrepancy via mental transformations (Vessey et al., 2006). As working memory is capacity limited, the magnitude of mental transformation or amount of working memory required is one predictor of reaction times and errors.

**Table 5:** Decision-making components that the reviewed studies evaluated the cognitive fit of

Cognitive fit examined	Studies
Visualization - > task	Dennis and Carte (1998); Grounds et al. (2017); Huang et al. (2006); Nadav-Greenberg et al. (2008); Smelcer and Carmel (1997); Vessey and Galletta (1991); Zhu and Watts (2010)
Visualization - > primed schema	Tversky et al. (2012)
Visualization - > learned schema	Feeney et al. (2000); Gattis and Holyoak (1996); Joslyn and LeClerc (2013)

**Fig. 13:**
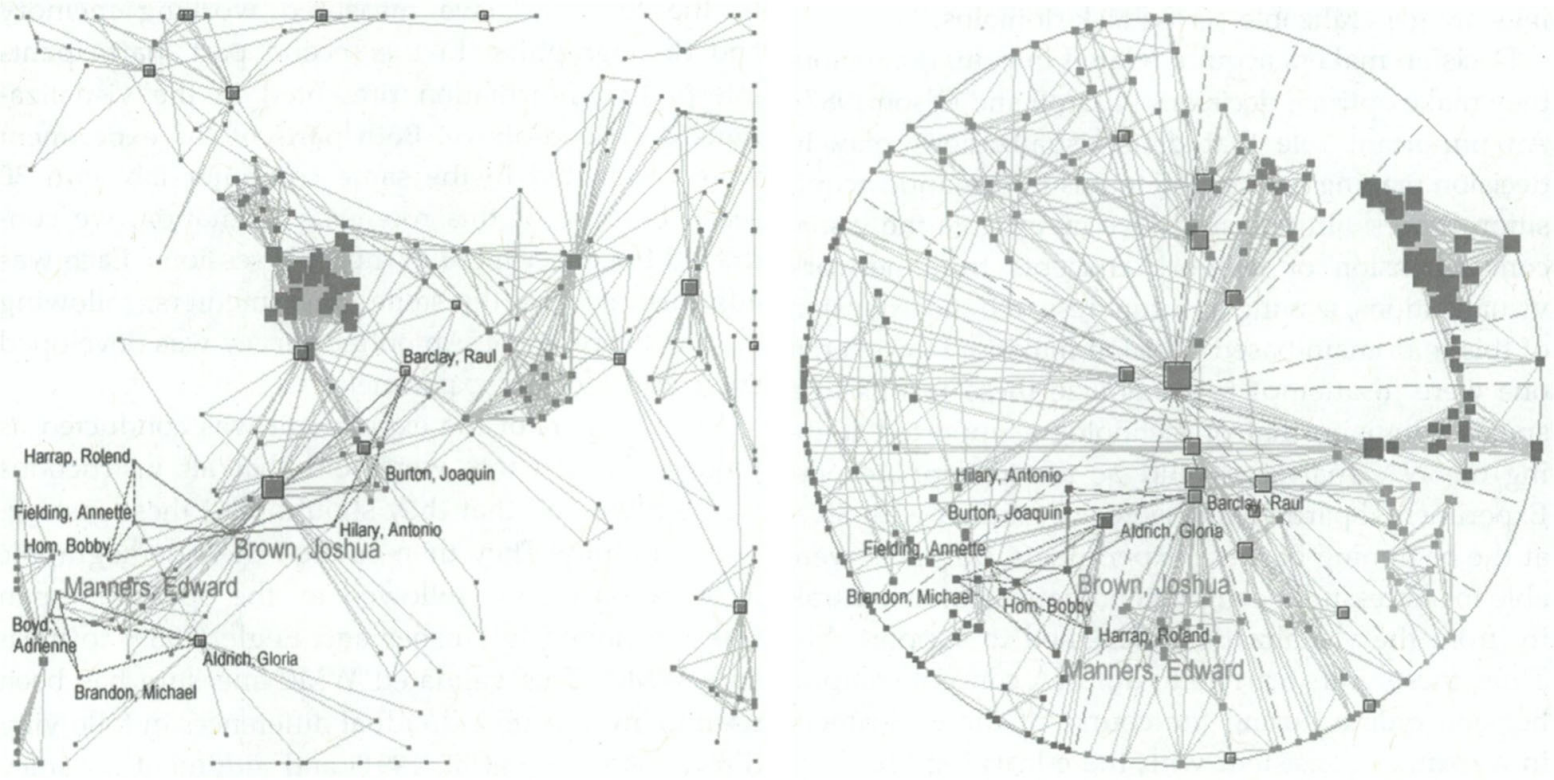
Examples of social media network diagrams from Zhu and Watts (2010). The authors argue that the figure on the *right* is more aligned with the task of identifying the most interconnected nodes than the figure on the *left*

Direct evidence for this claim comes from work demonstrating that cognitive fit differentially influenced the performance of individuals with more and less working memory capacity (Zhu & Watts, 2010). The task was to identify which two nodes in a social media network diagram should be removed to disconnect the maximal number of nodes. As predicted by cognitive fit theory, when the visualization did not facilitate the task (Fig. 13 left), participants with less working memory capacity were slower than those with more working memory capacity. However, when the visualization aligned with the task (Fig. 13 right), there was no difference in performance. This work suggests that when there is misalignment between the visualization and a decision-making process, people with more working memory capacity have the resources to resolve the conflict, while those with less resources show performance degradations.^2^ Other work only found a modest relationship between working memory capacity and correct interpretations of high and low temperature forecast visualizations (Grounds et al., 2017), which suggests that, for some visualizations, viewers utilize little working memory.

**Fig. 14:**
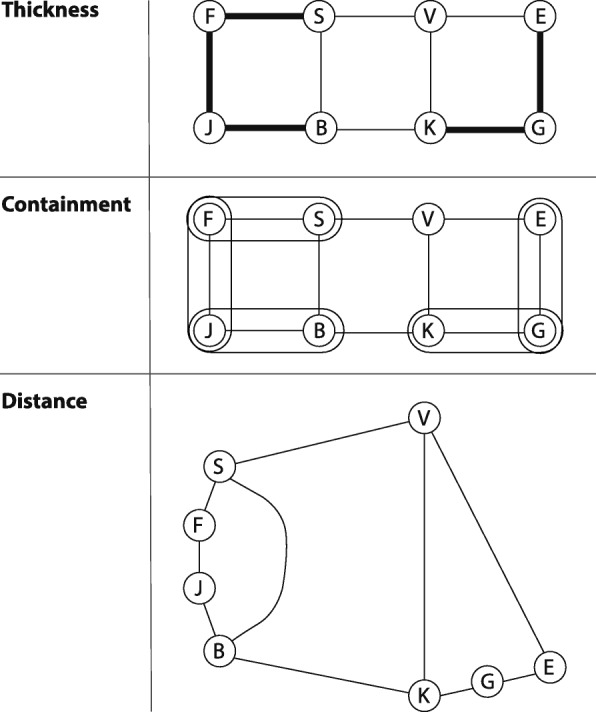
Example of stimuli from Tversky et al. (2012) showing three types of encoding techniques for connections between nodes (thickness, containment, and distance). Participants were asked to select routes between nodes with different descriptions of the visualizations. Redrawn from “Representing category and continuum: Visualizing thought” by B. Tversky, J. Corter, L. Yu, D. Mason, and J. Nickerson. In *Diagrams 2012* (p. 27), P. Cox, P. Rodgers, and B. Plimmer (Eds.), 2012, Berlin Heidelberg: Springer-Verlag

As illustrated in our model, working memory can be recruited to aid all stages of the decision-making process except bottom-up attention. Work that examines cognitive fit theory provides indirect evidence that working memory is required to resolve conflicts in the schema matching and a decision-making component. For example, one way that a mismatch between a viewer’s mental schema and visualization can arise is when the viewer uses a schema that is not optimal for the task. Tversky, Corter, Yu, Mason, and Nickerson (2012) primed participants to use different schemas by describing the connections in Fig. 14 in terms of either transfer speed or security levels. Participants then decided on the most efficient or secure route for information to travel between computer nodes with either a visualization that encoded data using the thickness of connections, containment, or physical distance (see Fig. 14). Tversky et al. (2012) found that when the links were described based on their information transfer speed, thickness and distance visualizations were the most effective—suggesting that the speed mental schema was most closely matched to the thickness and distance visualizations, whereas the speed schema required mental transformations to align with the containment visualization. Similarly, the thickness and containment visualizations outperformed the distance visualization when the nodes were described as belonging to specific systems with different security levels. This work and others (Feeney, Hola, Liversedge, Findlay, & Metcalf, 2000; Gattis & Holyoak, 1996; Joslyn & LeClerc, 2013; Smelcer & Carmel, 1997) provides indirect evidence that gratuitous realignment between mental schema and the visualization can be error-prone and visualization designers should work to reduce the number of transformations required in the decision-making process.

**Fig. 15:**
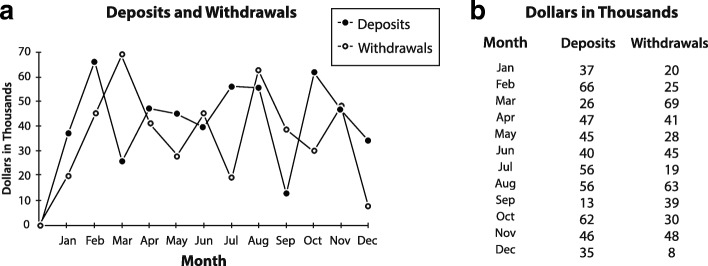
Examples of stimuli from Vessey and Galletta (1991) depicting deposits and withdraw amounts over the course of a year with a graph (**a**) and table (**b**). Participants completed either a spatial or textual task with the chart or table. Redrawn from “Cognitive fit: An empirical study of information acquisition” by I. Vessey, and D. Galletta. 1991, *Information systems research, 2*(1), 72–73. Copyright 1991 by “INFORMS”

Researchers from multiple domains have also documented cases of misalignment between the task, or conceptual question, and the visualization. For example, Vessey and Galletta (1991) found that participants completed a financial-based task faster when the visualization they chose (graph or table, see Fig. 15) matched the task (spatial or textual). For the spatial task, participants decided which month had the greatest difference between deposits and withdrawals. The textual or symbolic tasks involved reporting specific deposit and withdrawal amounts for various months. The authors argued that when there is a mismatch between the task and visualization, the additional transformation accounts for the increased time taken to complete the task (Vessey & Galletta, 1991) (see also Dennis & Carte, 1998; Huang et al., 2006), which likely takes place in the inference process of our proposed model.

The aforementioned studies provide direct (Zhu & Watts, 2010) and indirect (Dennis & Carte, 1998; Feeney et al., 2000; Gattis & Holyoak, 1996; Huang et al., 2006; Joslyn & LeClerc, 2013; Smelcer & Carmel, 1997; Tversky et al., 2012; Vessey & Galletta, 1991) evidence that Type 2 processing recruits working memory to resolve misalignment between decision-making processes and the visualization that arise from default Type 1 processing. These examples of Type 2 processing using working memory to perform effortful mental computations are consistent with the assertions of Evans and Stanovich (2013) that Type 2 processes enact goal directed complex processing. However, it is not clear from the reviewed work how *exactly* the visualization and decision-making components are matched. Newman and Scholl (2012) propose that we match the schema and visualization based on the similarities between the salient visual features, although this proposal has not been tested. Further, work that assesses cognitive fit in terms of the visualization and task only examines the alignment of broad categories (i.e., spatial or semantic). Beyond these broad classifications, it is not clear how to predict if a task and visualization are aligned. In sum, there is not a sufficient cross-disciplinary theory for *how* mental schemas and tasks are matched to visualizations. However, it is apparent from the reviewed work that Type 2 processes (requiring working memory) can be recruited during the schema matching and inference processes.

### Either type 1 and/or 2

#### Knowledge-driven processing

In a review of map-reading cognition, Lobben (2004) states, “…research should focus not only on the needs of the map reader but also on their map-reading skills and abilities” (p. 271). In line with this statement, the final cross-domain finding is that the effects of knowledge can interact with the affordances or biases inherent in the visualization method. Knowledge may be held temporally in working memory (Type 2), held in long-term knowledge but effortfully used (Type 2), or held in long-term knowledge but automatically applied (Type 1). As a result, knowledge-driven processing can involve either Type 1 or Type 2 processes.

Both short- and long-term knowledge can influence visualization affordances and biases. However, it is difficult to distinguish whether Type 2 processing is using significant working memory capacity to temporarily hold knowledge or if participants have stored the relevant knowledge in long-term memory and processing is more automatic. Complicating the issue, knowledge stored in long-term memory can influence decision making with visualizations using both Type 1 and 2 processing. For example, if you try to remember Pythagorean’s Theorem, which you may have learned in high school or middle school, you may recall that a^2^ + b^2^ = c^2^, where c represents the length of the hypotenuse and a and b represent the lengths of the other two sides of a triangle. Unless you use geometry regularly, you likely had to strenuously search in long-term memory for the equation, which is a Type 2 process and requires significant working memory capacity. In contrast, if you are asked to recall your childhood phone number, the number might automatically come to mind with minimal working memory required (Type 1 processing).

In this section, we highlight cases where knowledge either influenced decision making with visualizations or was present but did not influence decisions (see Table 6 for the type of knowledge examined in each study). These studies are organized based on how much time the viewers had to incorporate the knowledge (i.e. short-term instructions and long-term individual differences in abilities and expertise), which may be indicative of where the knowledge is stored. However, many factors other than time influence the process of transferring knowledge by working memory capacity to long-term knowledge. Therefore, each of the studies cited in this section could be either Type 1, Type 2, or both types of processing.

**Table 6:** Type of knowledge examined in each study

Knowledge	Studies
Short-term training, instructions	Boone et al. (in press); Shen et al. (2012)
Individual differences	Galesic and Garcia-Retamero (2011); Galesic et al. (2009) Keller et al. (2009) Okan et al. (2015); Okan, Garcia-Retamero, Galesic, and Cokely (2012); Okan, Garcia-Retamero, Cokely, and Maldonado (2012); Okan, Garcia-Retamero, Galesic, and Cokely (2012); Reyna et al. (2009); Rodríguez et al. (2013)
Semester-long course	Lee and Bednarz (2009)
Experts	Belia et al. (2005); Riveiro (2016); St. John et al. (2001)

**Fig. 16:**
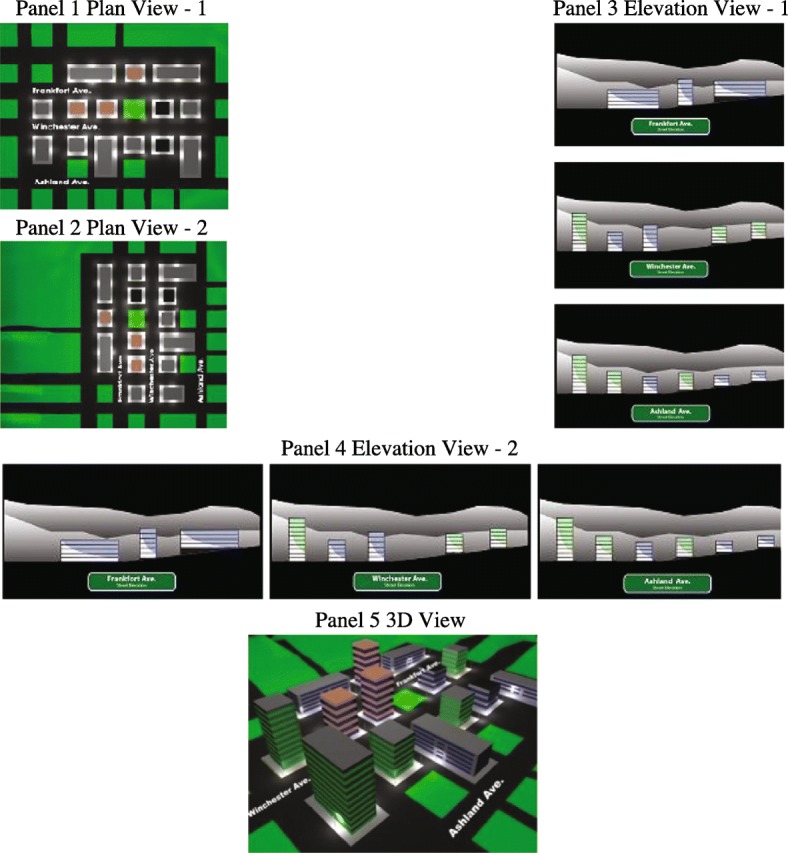
Example of different types of view orientations used by examined by Bailey et al. (2007). Participants selected one of these visualizations and then used their selection to make judgments including identifying safe passageways, determining appropriate locations for firefighters, and identifying suspicious locations based on the height of buildings. The panels correspond to the conditions in the original study

One example of participants using short-term knowledge to override a familiarity bias comes from work by Bailey, Carswell, Grant, and Basham (2007) (see also Shen, Carswell, Santhanam, & Bailey, 2012). In a complex geospatial task for which participants made judgments about terrorism threats, participants were more likely to select familiar map-like visualizations rather than ones that would be optimal for the task (see Fig. 16) (Bailey et al., 2007). Using the same task and visualizations, Shen et al. (2012) showed that users were more likely to choose an efficacious visualization when given training concerning the importance of cognitive fit and effective visualization techniques. In this case, viewers were able to use knowledge-driven processing to improve their performance. However, Joslyn and LeClerc (2013) found that when participants viewed temperature uncertainty, visualized as error bars around a mean temperature prediction, they incorrectly believed that the error bars represented high and low temperatures. Surprisingly, participants maintained this belief despite a key, which detailed the correct way to interpret each temperature forecast (see also Boone et al., in press). The authors speculated that the error bars might have matched viewers’ mental schema for high- and low-temperature forecasts (stored in long-term memory) and they incorrectly utilized the high-/low-temperature schema rather than incorporating new information from the key. Additionally, the authors propose that because the error bars were visually represented as discrete values, that viewers may have had difficulty reimagining the error bars as points on a distribution, which they term a *deterministic construal error* (Joslyn & LeClerc, 2013). Deterministic construal visual-spatial biases may also be one of the sources of misunderstanding of the Cone of Uncertainty (Padilla, Ruginski et al., 2017; Ruginski et al., 2016). A notable difference between these studies and the work of Shen et al. (2012) is that Shen et al. (2012) used instructions to correct a familiarity bias, which is a cognitive bias originally documented in the decision-making literature that is not based on the visual elements in the display. In contrast, the biases in Joslyn and LeClerc (2013) were visual-spatial biases. This provides further evidence that visual-spatial biases may be a unique category of biases that warrant dedicated exploration, as they are harder to influence with knowledge-driven processing.

**Fig. 17:**
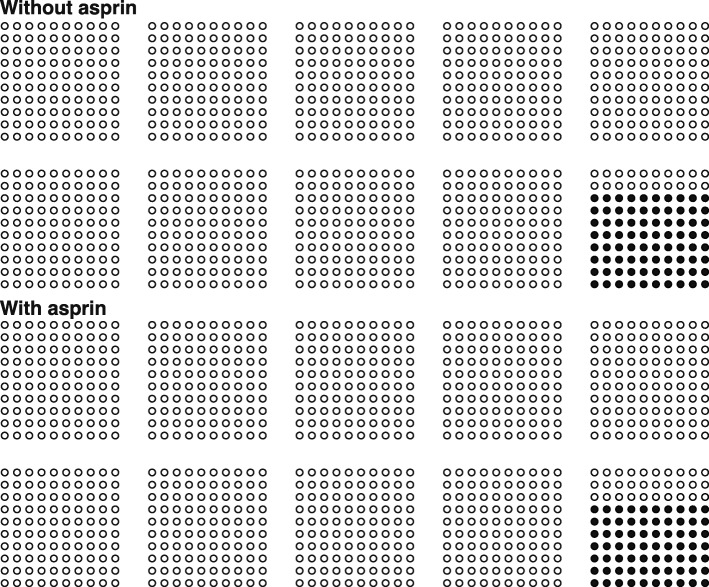
Example of stimuli used by Galesic et al. (2009) in a study demonstrating that natural frequency visualizations can help individuals overcome less numeracy. Participants completed three medical scenario tasks using similar visualizations as depicted here, in which they were asked about the effects of aspirin on risk of stroke or heart attack and about a hypothetical new drug. Redrawn from “Using icon arrays to communicate medical risks: overcoming less numeracy” by M. Galesic, R. Garcia-Retamero, and G. Gigerenzer. 2009, *Health Psychology, 28*(2), 210

Regarding longer-term knowledge, there is substantial evidence that individual differences in knowledge impact decision making with visualizations. For example, numerous studies document the benefit of visualizations for individuals with less health literacy, graph literacy, and numeracy (Galesic & Garcia-Retamero, 2011; Galesic, Garcia-Retamero, & Gigerenzer, 2009; Keller, Siegrist, & Visschers, 2009; Okan, Galesic, & Garcia-Retamero, 2015; Okan, Garcia-Retamero, Cokely, & Maldonado, 2012; Okan, Garcia-Retamero, Galesic, & Cokely, 2012; Reyna, Nelson, Han, & Dieckmann, 2009; Rodríguez et al., 2013). Visual depictions of health data are particularly useful because health data often take the form of probabilities, which are unintuitive. Visualizations inherently illustrate probabilities (i.e. 10%) as natural frequencies (i.e. 10 out of 100), which are more intuitive (Hoffrage & Gigerenzer, 1998). Further, by depicting natural frequencies visually (see example in Fig. 17), viewers can make perceptual comparisons rather than mathematical calculations. This dual benefit is likely the reason visualizations produce facilitation for individuals with less health literacy, graph literacy, and numeracy.

These studies are good examples of how designers can create visualizations that capitalize on Type 1 processing to help viewers accurately make decisions with complex data even when they lack relevant knowledge. Based on the reviewed work, we speculate that well-designed visualizations that utilize Type 1 processing to intuitively illustrate task-relevant relationships in the data may be particularly beneficial for individuals with less numeracy and graph literacy, even for simple tasks. However, poorly designed visualizations that require superfluous mental transformations may be detrimental to the same individuals. Further, individual differences in expertise, such as graph literacy, which have received more attention in healthcare communication (Galesic & Garcia-Retamero, 2011; Nayak et al., 2016; Okan et al., 2015; Okan, Garcia-Retamero, Cokely, & Maldonado, 2012; Okan, Garcia-Retamero, Galesic, & Cokely, 2012; Rodríguez et al., 2013), may play a large role in how viewers complete even simple tasks in other domains such as map-reading (Kinkeldey et al., 2017).

Less consistent are findings on how more experienced users incorporate knowledge acquired over longer periods of time to make decisions with visualizations. Some research finds that students’ decision-making and spatial abilities improved during a semester-long course on Geographic Information Science (GIS) (Lee & Bednarz, 2009). Other work finds that experts perform the same as novices (Riveiro, 2016), experts can exhibit visual-spatial biases (St. John et al., 2001) and experts perform more poorly than expected in their domain of visual expertise (Belia et al., 2005). This inconsistency may be due in part to the difficulty in identifying *when* and *if* more experienced viewers are automatically applying their knowledge or employing working memory. For example, it is unclear if the students in the GIS course documented by Lee and Bednarz (2009) developed automatic responses (Type 1) or if they learned the information and used working memory capacity to apply their training (Type 2).

**Fig. 18:**
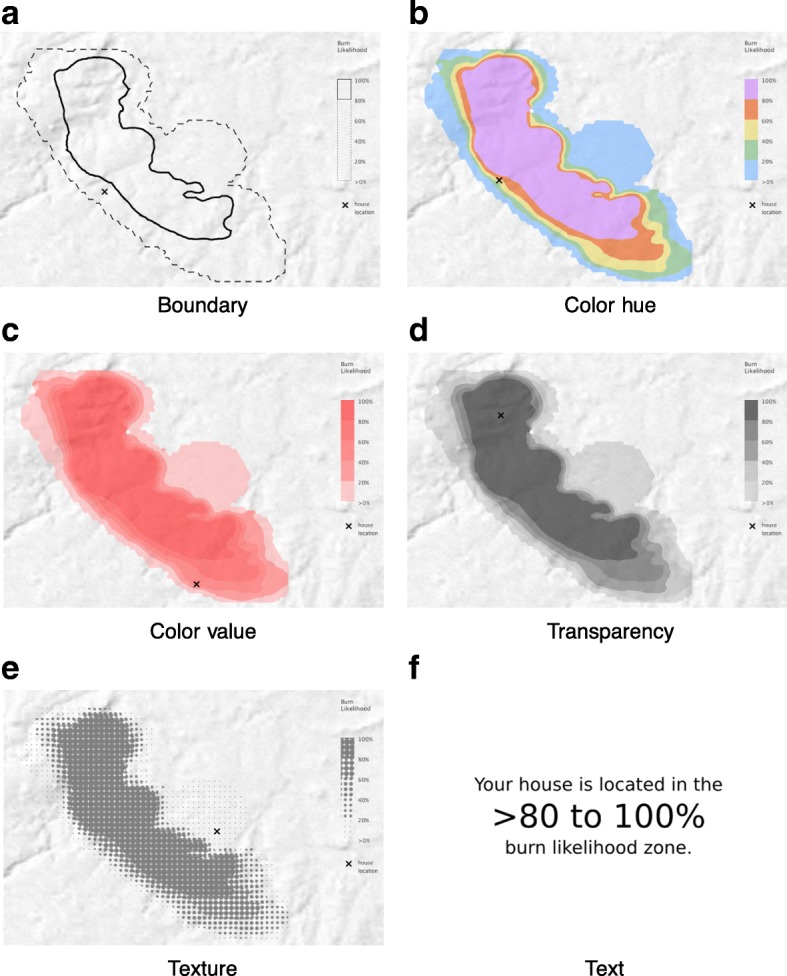
Example of multiple uncertainty visualization techniques for wildfire risk by Cheong et al. (2016). Participants were presented with a house location (indicated by an X), and asked if they would stay or leave based on one of the wildfire hazard communication techniques shown here. The panels correspond to the conditions in the original study

Cheong et al. (2016) offer one way to gauge how performance may change when one is forced to use Type 1 processing, but then allowed to use Type 2 processing. In a wildfire task using multiple depictions of uncertainty (see Fig. 18), Cheong et al. (2016) found that the type of uncertainty visualization mattered when participants had to make fast Type 1 decisions (5 s) about evacuating from a wildfire. But when given sufficient time to make Type 2 decisions (30 s), participants were not influenced by the visualization technique (see also Wilkening & Fabrikant, 2011).

Interesting future work could limit experts’ time to complete a task (forcing Type 1 processing) and then determine if their judgments change when given more time to complete the task (allowing for Type 2 processing). To test this possibility further, a dual-task paradigm could be used such that experts’ working memory capacity is depleted by a difficult secondary task that also required working memory capacity. Some examples of secondary tasks in a dual-task paradigm include span tasks that require participants to remember or follow patterns of information, while completing the primary task, then report the remembered or relevant information from the pattern (for a full description of theoretical bases for a dual-task paradigm see Pashler, 1994). To our knowledge, only one study has used a dual-task paradigm to evaluate cognitive load of a visualization decision-making task (Bandlow et al., 2011). However, a growing body of research on other domains, such as wayfinding and spatial cognition, demonstrates the utility of using dual-task paradigms to understand the types of working memory that users employ for a task (Caffò, Picucci, Di Masi, & Bosco, 2011; Meilinger, Knauff, & Bülthoff, 2008; Ratliff & Newcombe, 2005; Trueswell & Papafragou, 2010).

**Fig. 19:**
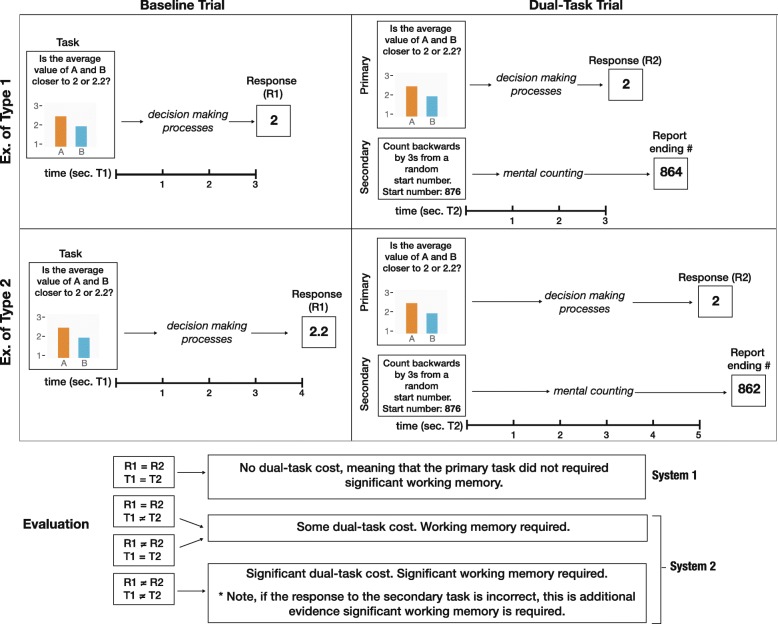
A diagram of a dual-tasking experiment is shown using the same task as in Fig. 5. Responses resulting from Type 1 and 2 processing are illustrated. The dual-task trial illustrates how to place additional load on working memory capacity by having the participant perform a demanding secondary task. The impact of the secondary task is illustrated for both time and accuracy. Long-term memory can influence all components and processes in the model either via pre-attentive processes or by conscious application of knowledge

*Span tasks* are examples of spatial or verbal secondary tasks, which include remembering the orientations of an arrow (taxes visual-spatial memory, (Shah & Miyake, 1996) or counting backward by 3 s (taxes verbal processing and short-term memory) (Castro, Strayer, Matzke, & Heathcote, 2018). One should expect more interference if the primary and secondary tasks recruit the same processes (i.e. visual-spatial primary task paired with a visual-spatial memory span task). An example of such an experimental design is illustrated in Fig. 19. In the dual-task trial illustrated in Fig. 19, if participants responses are as fast and accurate as the baseline trial then participants are likely not using significant amounts of working memory capacity for that task. If the task does require significant working memory capacity, then the inclusion of the secondary task should increase the time taken to complete the primary task and potentially produce errors in both the secondary and primary tasks. In visualization decision-making research, this is an open area of exploration for researchers and designers that are interested in understanding how working memory capacity and a dual-process account of decision making applies to their visualizations and application domains.

In sum, this section documents cases where knowledge-driven processing does and does not influence decision making with visualizations. Notably, we describe numerous studies where well-designed visualizations (capitalizing on Type 1 processing) focus viewers’ attention on task-relevant relationships in the data, which improves decision accuracy for individuals with less developed health literacy, graph literacy, and numeracy. However, the current work does not test how knowledge-driven processing maps on to the dual-process model of decision making. Knowledge may be held temporally by working memory capacity (Type 2), held in long-term knowledge but strenuously utilized (Type 2), or held in long-term knowledge but automatically applied (Type 1). More work is needed to understand if a dual-process account of decision making accurately describes the influence of knowledge-driven processing on decision making with visualizations. Finally, we detailed an example of a dual-task paradigm as one way to evaluate if viewers are employing Type 1 processing.

## Review summary

Throughout this review, we have provided significant direct and indirect evidence that a dual-process account of decision making effectively describes prior findings from numerous domains interested in visualization decision making. The reviewed work provides support for specific processes in our proposed model including the influences of working memory, bottom-up attention, schema matching, inference processes, and decision making. Further, we identified key commonalities in the reviewed work relating to Type 1 and Type 2 processing, which we added to our proposed visualization decision-making model. The first is that utilizing Type 1 processing, visualizations serve to direct participants’ bottom-up attention to specific information, which can be either beneficial or detrimental for decision making (Fabrikant et al., 2010; Fagerlin et al., 2005; Hegarty et al., 2010; Hegarty et al., 2016; Padilla, Ruginski et al., 2017; Ruginski et al., 2016; Schirillo & Stone, 2005; Stone et al., 1997; Stone et al., 2003; Waters et al., 2007). Consistent with assertions from cognitive science and scientific visualization (Munzner, 2014), we propose that visualization designers should identify the critical information needed for a task and use a visual encoding technique that directs participants’ attention to this information. We encourage visualization designers who are interested in determining which elements in their visualizations will likely attract viewers’ bottom-up attention, to see the Itti et al. (1998) saliency model, which has been validated with eye-tracking measures (for implementation of this model along with Matlab code see Padilla, Ruginski et al., 2017). If deliberate effort is not made to capitalize on Type 1 processing by focusing the viewer’s attention on task-relevant information, then the viewer will likely focus on distractors via Type 1 processing, resulting in poor decision outcomes.

A second cross-domain finding is the introduction of a new concept, *visual-spatial biases*, which can also be both beneficial and detrimental to decision making. We define this term as a bias that elicits heuristics, which is a direct result of the visual encoding technique. We provide numerous examples of visual-spatial biases across domains (for implementation of this model along with Matlab code, see Padilla, Ruginski et al., 2017). The novel utility of identifying visual-spatial biases is that they potentially arise early in the decision-making process during bottom-up attention, thus influencing the entire downstream process, whereas standard heuristics do not exclusively occur at the first stage of decision making. This possibly accounts for the fact that visual-spatial biases have proven difficult to overcome (Belia et al., 2005; Grounds et al., 2017; Joslyn & LeClerc, 2013; Liu et al., 2016; McKenzie et al., 2016; Newman & Scholl, 2012; Padilla, Ruginski et al., 2017; Ruginski et al., 2016). Work by Tversky (2011) presents a taxonomy of visual-spatial communications that are intrinsically related to thought, which are likely the bases for visual-spatial biases.

We have also revealed cross-domain findings involving Type 2 processing, which suggest that if there is a mismatch between the visualization and a decision-making component, working memory is used to perform corrective mental transformations. In scenarios where the visualization is aligned with the mental schema and task, performance is fast and accurate (Joslyn & LeClerc, 2013). The types of mismatches observed in the reviewed literature are likely both domain-specific and domain-general. For example, situations where viewers employ the correct graph schema for the visualization, but the graph schema does not align with the task, are likely domain-specific (Dennis & Carte, 1998; Frownfelter-Lohrke, 1998; Gattis & Holyoak, 1996; Huang et al., 2006; Joslyn & LeClerc, 2013; Smelcer & Carmel, 1997; Tversky et al., 2012). However, other work demonstrates cases where viewers employ a graph schema that does not match the visualization, which is likely domain-general (e.g. Feeney et al., 2000; Gattis & Holyoak, 1996; Tversky et al., 2012). In these cases, viewers could accidentally use the wrong graph schema because it appears to match the visualization or they might not have learned a relevant schema. The likelihood of viewers making attribution errors because they do not know the corresponding schema increases when the visualization is less common, such as with uncertainty visualizations. When there is a mismatch, additional working memory is required resulting in increased time taken to complete the task and in some cases errors (e.g. Joslyn & LeClerc, 2013; McKenzie et al., 2016; Padilla, Ruginski et al., 2017). Based on these findings, we recommend that visualization designers should aim to create visualizations that most closely align with a viewer’s mental schema and task. However, additional empirical research is required to understand the nature of the alignment processes, including the exact method we use to mentally select a schema and the classifications of tasks that match visualizations.

The final cross-domain finding is that knowledge-driven processes can interact or override effects of visualization methods. We find that short-term (Dennis & Carte, 1998; Feeney et al., 2000; Gattis & Holyoak, 1996; Joslyn & LeClerc, 2013; Smelcer & Carmel, 1997; Tversky et al., 2012) and long-term knowledge acquisition (Shen et al., 2012) can influence decision making with visualizations. However, there are also examples of knowledge having little influence on decisions, even when prior knowledge could be used to improve performance (Galesic et al., 2009; Galesic & Garcia-Retamero, 2011; Keller et al., 2009; Lee & Bednarz, 2009; Okan et al., 2015; Okan, Garcia-Retamero, Cokely, & Maldonado, 2012; Okan, Garcia-Retamero, Galesic, & Cokely, 2012; Reyna et al., 2009; Rodríguez et al., 2013). We point out that prior knowledge seems to have more of an effect on non-visual-spatial biases, such as a familiarity bias (Belia et al., 2005; Joslyn & LeClerc, 2013; Riveiro, 2016; St. John et al., 2001), which suggests that visual-spatial biases may be closely related to bottom-up attention. Further, it is unclear from the reviewed work when knowledge switches from relying on working memory capacity for application to automatic application. We argue that Type 1 and 2 processing have unique advantages and disadvantages for visualization decision making. Therefore, it is valuable to understand which process users are applying for specific tasks in order to make visualizations that elicit optimal performance. In the case of experts and long-term knowledge, we propose that one interesting way to test if users are utilizing significant working memory capacity is to employ a dual-task paradigm (illustrated in Fig. 19). A dual-task paradigm can be used to evaluate the amount of working memory required and compare the relative working memory required between competing visualization techniques.

We have also proposed a variety of practical recommendations for visualization designers based on the empirical findings and our cognitive framework. Below is a summary list of our recommendations along with relevant section numbers for reference:

- Identify the critical information needed for a task and use a visual encoding technique that directs participants’ attention to this information (“Bottom-up attention” section);
- To determine which elements in a visualization will likely attract viewers’ bottom-up attention try employing a saliency algorithm (see Padilla, Quinan, et al., 2017) (see “Bottom-up attention”);
- Aim to create visualizations that most closely align with a viewer’s mental schema and task demands (see “Visual-Spatial Biases”);
- Work to reduce the number of transformations required in the decision-making process (see "Cognitive fit");
- To understand if a viewer is using Type 1 or 2 processing employ a dual-task paradigm (see Fig. 19);
- Consider evaluating the impact of individual differences such as graphic literacy and numeracy on visualization decision making.

## Conclusions

We use visual information to inform many important decisions. To develop visualizations that account for real-life decision making, we must understand how and why we come to conclusions with visual information. We propose a dual-process cognitive framework expanding on visualization comprehension theory that is supported by empirical studies to describe the process of decision making with visualizations. We offer practical recommendations for visualization designers that take into account human decision-making processes. Finally, we propose a new avenue of research focused on the influence of visual-spatial biases on decision making.

## Footnotes

1 Dual-process theory will be described in greater detail in next section.

2 It should be noted that in some cases the activation of Type 2 processing should improve decision accuracy. More research is needed that examines cases where Type 2 could improve decision performance with visualizations.
