# Correction to: Decision making with visualizations: a cognitive framework across disciplines

**DOI:** 10.1186/s41235-018-0126-3

**Published:** 2018-09-02

**Authors:** Lace M. Padilla, Sarah H. Creem-Regehr, Mary Hegarty, Jeanine K. Stefanucci

**Affiliations:** 1 Northwestern University, Evanston, USA; 2 Department of Psychology, University of Utah, 380 S. 1530 E., Room 502, Salt Lake City, UT 84112 USA; 3 Department of Psychology, University of California–Santa Barbara, Santa Barbara, USA

## Correction

The original article (Padilla et al., 2018) contained a formatting error in Table 2; this has now been corrected with the appropriate boxes marked clearly.

**Table 2:** Overview of the four cross-domain findings along with the type of processing that they reflect

		Evidence for Type		
	Cross-domain finding	1	2	Either
1	Visualizations direct viewers’ *bottom-up attention*, which can both help and hinder decision making.	X		
2	The visual encoding technique gives rise to *visual-spatial biases*.	X		
3	Visualizations that have greater *cognitive fit* produce faster and more effective decisions.		X	
4	*Knowledge-driven processes* can interact with the effects of the encoding technique.			X

The italicised words correspond to section titles

## References

[CR1] Padilla LM (2018). Decision making with visualizations: a cognitive framework across disciplines. Cognitive Research: Principles and Implications.

